# Associations between Physiological Biomarkers and Psychosocial Measures of Pregnancy-Specific Anxiety and Depression with Support Intervention

**DOI:** 10.3390/ijerph18158043

**Published:** 2021-07-29

**Authors:** Karen L. Weis, Tony T. Yuan, Katherine C. Walker, Thomas F. Gibbons, Wenyaw Chan

**Affiliations:** 1 School of Nursing and Health Professions, University of the Incarnate Word, 4301 Broadway, CPO #300, San Antonio, TX 78209, USA; kcwalker@uiwtx.edu; 2 Science and Technology, 59th Medical Wing, 1632 Nellis St. Bldg. 5406, JBSA-Lackland, TX 78236, USA; tony.t.yuan.civ@mail.mil (T.T.Y.); thomas.f.gibbons6.civ@mail.mil (T.F.G.); 3 Department of Biostatistics and Data Science, School of Public Health, University of Texas-Health Science Center at Houston, 1200 Pressler St., Houston, TX 77030, USA; Wenyaw.Chan@uth.tmc.edu

**Keywords:** pregnancy, prenatal anxiety, depression, cytokines, intervention, military

## Abstract

Stress and anxiety significantly impact the hypothalamic–pituitary axis, and in pregnancy, the subsequent maternal–fetal response can lead to poor outcomes. The objective of this study was to assess the association between psychosocial measures of pregnancy-specific anxiety and physiologic inflammatory responses. Specifically, to determine the effectiveness of the Mentors Offering Maternal Support (M-O-M-S^TM^) program to reduce psychosocial anxiety and associated inflammatory response. In conjunction with measures of pregnancy-specific anxiety and depression, serum biomarkers (IL-2, IL-6, IL-10, IL1-B, TNF-α, CRH, CRP, and cortisol) were analyzed for each trimester throughout pregnancy. Results demonstrated that women receiving the M-O-M-S^TM^ intervention had longitudinally sustained lower TNF-α/IL-10 ratios than the control group, and it was significantly associated with psychosocial measures of anxiety, specifically for *fears of labor* and *spouse/partner relationships*. Additionally, the anxiety of *spouse/partner relationships* was significantly associated with IL-6/IL-10 ratios. The findings highlight the important counter-regulatory relationship between anti- and pro-inflammatory cytokines and provide insight into the distinct physiologic responses to pregnancy-specific anxiety with early prenatal intervention.

## 1. Introduction

Maternal adaptive responses to psychosocial stress and anxiety during pregnancy are inherently complex and critical in maintaining a unique immune-privileged environment that influences birth outcomes. Specifically, the hypothalamic–pituitary–adrenal (HPA) axis stress response and the subsequent production of glucocorticoids is delicately balanced throughout the pregnancy to ensure healthy outcomes [1]. As the placenta is a stress-sensitive organ, any modulation of corticotrophin-releasing hormone (CRH) may augment labor [2]. Increased, normal, or decreased levels of CRH concentration have been associated with preterm, term, or post-term labor, respectively. In addition to changes in stress response, the normal immune response in pregnant mothers progressively shifts from a cell-mediated, pro-inflammatory, Th1 response to a humoral, anti-inflammatory, Th2 response [3]. The balance between Th1 and Th2 response and the interaction of the associated pro- and anti-inflammatory cytokines, particularly TNF-α, IL-1β, IL-6, and IL-10, are important in the development and maintenance of a normal pregnancy [4,5].

Both prospective and retrospective pregnancy studies in humans and animals suggest that psychosocial stress and anxiety can profoundly affect immune response, leading to complications such as preterm birth, preeclampsia, and poor birth outcomes [6,7]. However, pregnancy anxiety and stress-associated physiological mechanisms contributing to poor birth and infant outcomes are not well-established and inconsistent across the literature. Gelman et al. demonstrated that women with severe anxiety had higher levels of both Th1 and Th2 cytokines in the 3rd trimester than those without any reported anxiety or depression, and those with both severe depression and anxiety had the highest concentration of cytokines [8]. Specifically, women with elevated stress scores had higher levels of pro-inflammatory cytokines IL-6 and TNF-α, and lower levels of the anti-inflammatory marker IL-10 [9]. Conversely, studies have found positive correlations for IL-12, IL-13, and IL-10 to pregnancy-related anxiety with no associations with IL-6 or TNF-α [10]. Additionally, an analysis of amniotic fluid obtained through transabdominal amniocentesis at 16–18 weeks, found significant differences in the levels of IL-1α, Il-1β, IL-4, IL-6, and IL-8, with no differences in TNF-α, for women who delivered preterm versus those at term [11].

Psychosocial interventions and effective social support systems play critical roles in mitigating poor birth outcomes by effectively changing immunological status during pregnancy. Specifically, in two different studies comparing social support to inflammatory responses, women perceiving higher social support had lower serum levels of pro-inflammatory cytokines (IL-2, IL-5, and IL-6) and C-reactive protein [12,13]. Additionally, in the Giurgescu et al. study, women with lower levels of IL-10 had an increased incidence of preterm birth [12]. Clearly, the association between psychosocial measures and physiologic changes in pregnancy requires further consideration that includes longitudinal assessment of both measures and adjustment for known confounders. Furthermore, there is a need to evaluate perinatal interventions and their effectiveness to modulate both psychosocial and physiologic measures, and to link effects to birth outcomes. As such, the purpose of this study was to explore the association of psychosocial measures of pregnancy-specific anxiety and depressive symptoms with serum samples of pro- and anti-inflammatory markers for women receiving the early prenatal Mentors Offering Maternal Support (M-O-M-S™) program. The M-O-M-S™ program, (an early prenatal support intervention) was designed for military women (both active-duty and spouses of active-duty service members) as the stressors of military life and geographical separation from support systems are common. The findings from the program pilot and the randomized-controlled trials have been previously published [14,15,16]. Specifically, we tested the hypotheses that (i) psychosocial stress of pregnancy promotes production of pro-inflammatory cytokines and inhibits the production of anti-inflammatory cytokines, and (ii) participation in the M-O-M-S™ program ameliorates the psychosocial stress of pregnancy evidenced by decreased changes in both psychosocial and physiologic measures of stress and anxiety.

## 2. Methods

### 2.1. Study Design

This study is part of the M-O-M-S™ project sponsored by the TriService Nursing Research Program as a prospective, longitudinal investigation of immune, inflammatory response to pregnancy-specific anxiety and depressive symptoms in conjunction with the M-O-M-S™ intervention with IRB approval (IRB #377034).

### 2.2. Participants

All pregnant military beneficiaries (both active-duty women and spouses of active-duty service members) initiating obstetrical care from 20 June 2012 to 16 June 2015 were contacted regarding their interest in the M-O-M-S™ study and screened for eligibility. Women were considered eligible for inclusion in the study if they were (1) ≤12 weeks gestation at time of recruitment and consent, (2) at least 18 years old, (3) an active-duty pregnant woman or a pregnant spouse of an active-duty member of the United States Armed Forces, and (4) able to understand English. Exclusion criteria included: (1) multiple gestation, (2) diabetes mellitus requiring insulin, (3) thyroid disorders, (4) chronic renal or heart disease, and/or (5) history and treatment for asthma.

### 2.3. Data Collection

Once consented, women were assigned to the treatment (M-O-M-S™ program) or control (prenatal care without the M-O-M-S™) groups based on a computer-generated randomization pattern. The M-O-M-S™ intervention group attended eight one-hour sessions every other week starting in the first trimester. Each session’s content was focused on unique aspects related to pregnancy-specific anxiety previously identified and piloted [15,17,18]. A detailed explanation of the intervention session content has been previously reported [14].

At the first trimester, and at approximate 16-week, and 28-week routine lab draws, the participants completed study questionnaire booklets containing the psychological measures and the demographic information sheet prior to the laboratory blood draw. A maternal venous blood sample of one 10 mL serum separator tube was collected in addition to the routine obstetrical labs. Given all analytes being collected (IL-6, TNF-α, IL-1β, IL-2, IL-10, CRP, CRH, and cortisol) had some diurnal rhythm, data collection was encouraged from 10:00 a.m. to 12:00 p.m., with no requirement for fasting. The study samples were immediately centrifuged, aliquoted, and frozen at −80 °C for batch analysis by trimester.

### 2.4. Immunologic Assays

The samples for IL-6, TNF-α, IL-1β, IL-2, and IL-10 were analyzed in duplicate using a multiplex cytometric bead array (Milliplex, cat# HCYTOMAG-60K, MilliporeSigma, Burlington, VT, USA) and read on a Luminex MagPix (Luminex, Austin, TX, USA). Singleplex ELISAs for CRH (MyBiosource, cat # MBS731545, MyBiosource, San Diego, CA, USA), CRP (R&D Systems, cat # SCRP00, R&D Systems, Minneapolis, MN, USA), and cortisol (Diagnostic Automation, cat# 6101-15, Diagnostic Automation/Cortez Diagnostics, INC., Woodland Hills, LA, USA) were analyzed in duplicate and read on a BioTek Synergy H4 plate reader (BioTek, Winooski, VT, USA). All assays were performed in accordance with the manufacturer’s instructions.

### 2.5. Psychological Measures

Pregnancy-Specific Anxiety. Lederman’s Prenatal Self-Evaluation Questionnaire (PSEQ-SF), a 53-item, seven-scaled instrument measuring aspects of pregnancy anxiety related to *Acceptance of Pregnancy*, *Identification with a Motherhood Role*, *Preparation for Labor*, *Concerns for Well-Being of Self and Baby in Labor*, *Fear of Pain, Helplessness, and Loss of Control in Labor*, *Relationship with Mother*, and *Relationship with Spouse/Partner*, was given in each trimester of pregnancy. Each item is measured on a four-point Likert scale ranging from 1 (Not at All) to 4 (Very Much So), where higher aggregate scores indicate greater pregnancy-specific anxiety. The instrument has been used extensively both in the United States and internationally with good results. The Cronbach alpha coefficients for this population ranged from α = 0.70 to 0.94. Convergent and divergent construct validity for the items was ascertained with biochemical assessments linked to qualitative data and to other validated anxiety measures [18,19].

Symptoms of Depression. The *Edinburgh Postnatal Depression Scale* (EPDS), a 10-item instrument measuring the presence of symptoms indicative of possible depression or possible anxiety was given in each trimester of pregnancy. The instrument is validated for both prenatal and postnatal use with each item scored 0–3 and higher scores reflecting greater symptoms of depression [20,21]. Aggregate scores of ≥13 are considered a “positive” score requiring follow-up [22], and a score other than 0 for item 10 (risk of self-harm) requires immediate attention. As such, all participants with scores ≥ 13 were referred for evaluation by behavioral health, and those indicating feelings of self-harm were evaluated immediately within the obstetrical clinic and sent to the emergency department if necessary. The Cronbach alpha coefficients for this population ranged from α = 0.86 to 0.87 across the three trimesters.

### 2.6. Statistical Analysis

Longitudinal mixed-effect regression models were applied to examine the slope difference of each serum biomarker between treatment groups, age groups (<=25 vs. >25), and parity, separately as well as by subgroup. The outcome variables included IL-6, TNF-α, IL-1β, IL-2, IL-10, CRP, CRH, cortisol, the ratio of TNF-α and IL-10, and the ratio of IL-6 and IL-10. Similar mixed-effect models were used to investigate the longitudinal relationship between each biomarker and each anxiety and stress component. These analyses were conducted and compared between the groups of aforementioned variables—treatment, age, and parity. Comparisons of cytokine levels by age group and parity were selected because of findings reported for differences in cytokine levels by maternal age and parity [23,24,25,26]. Additionally, we had previously found parity to be the only significant variable within the model for *Preparation for Labor* anxiety. Participants who were nulliparous had significantly higher scores for this dimension of anxiety [14]. Parity was also a significant predictor of increased anxiety for *Acceptance of Pregnancy, Preparation for Labor, and Fear of Pain, Helplessness, and Loss of Control in Labor.* However, in this case the nulliparas had scores that were significantly lower for *Acceptance of Pregnancy* and higher scores for *Fear of Pain, Helplessness, and Loss of Control in Labor.* The correlation structure of the repeated observations of each dependent variable was assumed to follow an autoregressive model of order 1. Important advantages of using these longitudinal regression models for comparing the slopes include their capability to reduce bias due to heterogeneity of the initial value and consideration of the correlated structure of each dependent variable repeatedly measured over time.

## 3. Results

Three hundred and sixty-seven women were randomized to either the M-O-M-S™ intervention or control groups. Separately, women participating in the M-O-M-S™ study were recruited and consented for the biomarker component of the study. Fifty-seven women were consented, of which the majority completed all serum collection points and psychosocial measures (Table 1). Detailed descriptive statistics for all the psychosocial variables were provided in Weis, et al., 2017 [14]. Analysis of the overall sample found statistically significant increases from first to third trimester in IL-6, CRP, CRH, and cortisol. There were no significant differences for any of the biomarkers by parity. There was a statistically significant change longitudinally in IL-6 for women older than 25 years of age. However, the same differences were not reflected in women younger than 25 years. Descriptive statistics for all the maternal serum biomarkers by trimester are provided in Table 2.

**Figure 1:**
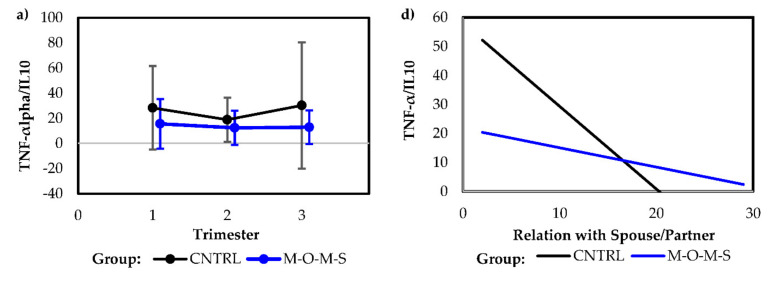

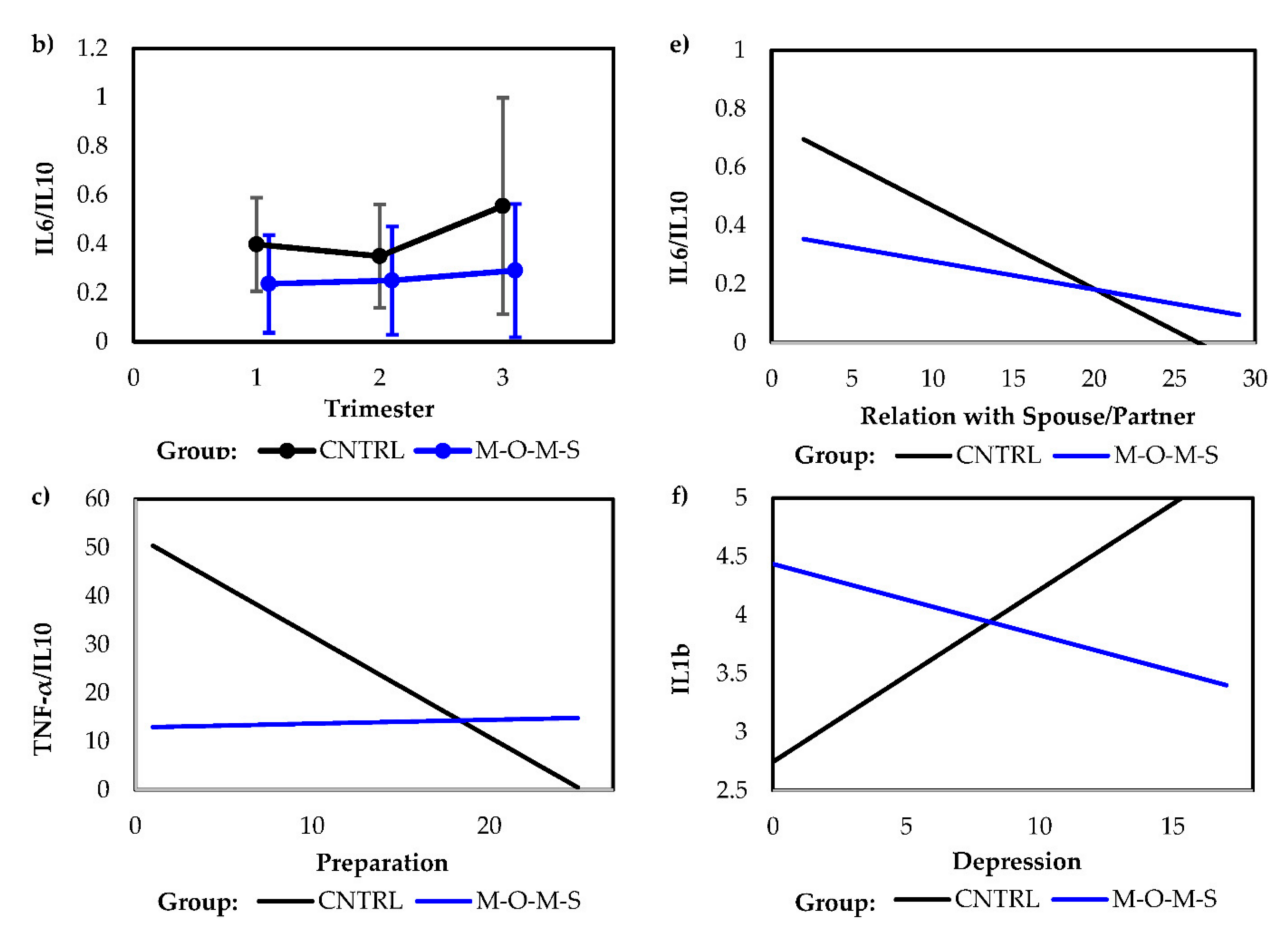
(**a**) Longitudinal comparison of TNF-alpha/IL-10 ratio for M-O-M-S^TM^ intervention and control groups. (**b**) Longitudinal comparison of IL-6/IL-10 ratio for M-O-M-S^TM^ intervention and control groups. (**c**) Longitudinal relationship between TNF-α/IL-10 ratio to reparation for Labor anxiety for M-O-M-S^TM^ intervention and control groups. (**d**) Longitudinal relationship between TNF-α/IL-10 ratio to Relationship with Spouse/Partner anxiety for M-O-M-S^TM^ intervention and control groups. (**e**) Longitudinal relationship between IL-6/IL-10 ratio to Relationship with Spouse/Partner anxiety for M-O-M-S^TM^ intervention and control groups. (**f**) Longitudinal relationship between IL1β to depressive symptoms for M-O-M-S^TM^ intervention and control groups.

In terms of the M-O-M-S™ intervention, participation demonstrated a longitudinally sustained lower TNF-α/IL-10 ratio than the control (*F*(1, 55) = 5.01, *p* = 0.03) (Figure 1a). Concurrently, there was a significant negative association for IL-10 in the control group (*F*(1, 55) = 6.59, *p* = 0.01) and for IL-6/IL-10 ratio (*F*(1, 55) = 11.78, *p* < 0.01) (Figure 1b). Comparisons to psychosocial measures of anxiety reflected a significant negative association between TNF-α/IL-10 ratio and *Preparation for Labor* (*p* = 0.03) (Figure 1c) and for *Relationship with Spouse/Partner* (*p* = 0.01) (Figure 1d) in pregnancy for the control group. After adjusting for the other, *Preparation for Labor* remained significant (*p* = 0.03) (Table 3). Similarly, the IL-6/IL-10 ratio reflected a negative association with anxiety related to *Relationship with Spouse/Partner* (*p* < 0.01) (Figure 1e). The IL-6/IL-10 ratio also reflected a higher value with a borderline significantly negative effect associated with *Identification with a Motherhood Role* (*p* = 0.07)*, Relationship with Mother* (*p* = 0.06), and *Preparation for Labor* (*p* = 0.07), a finding not reflected in the treatment group, which remained relatively constant across pregnancy. Closely aligned, the treatment group had an impressive increase, albeit borderline significant (*p* = 0.07), for IL-10 associated with *Identification with a Motherhood Role*, while the control group had a low, straight trajectory for IL-10. The control group also reflected a significant increase in IL-1β to *depressive symptoms* (*p* = 0.01) over that of the treatment group (Figure 1f).

## 4. Discussion

The current study provides longitudinal data for cytokine profiles in pregnancy with and without the addition of an early pregnancy, anxiety-reducing intervention. Notably, the results highlight the critical counter-regulatory relationship between the anti-inflammatory cytokine IL-10 and pro-inflammatory cytokines IL-6 and TNF-α. It is hypothesized that without the protective, regulatory function of IL-10, the risk of obstetric complications increases [5]. Consistent with previous literature reports [8], our results found that IL-6 and TNF-α were significantly important in their association with pregnancy anxiety and function as drivers of inflammation. Additionally, the results provided insight into the differences in distinct physiological, inflammatory responses to pregnancy-specific anxiety based on group assignment. Women receiving the M-O-M-S™ support intervention had a sustained, balanced ratio of pro- versus anti-inflammatory cytokines. Conversely, the control group demonstrated a significant shift in their cytokine pattern that reflects a non-balanced inflammatory physiological state and highlighted the counter regulatory role of IL-10. Additionally, the control group had a significant increase over that of the treatment group for the pro-inflammatory marker, IL-1β, associated with increased depressive symptoms. Remarkably, our data reflects physiologic changes associated with relatively slight variations in the anxiety and depression measures rather than severe anxiety or comorbid depression.

It is increasingly clear that maternal inflammatory responses have a strong association with poor pregnancy outcomes. Although pro-inflammatory cytokines are the most broadly understood for their significant effects on birth outcomes, the nuanced relationship with anti-inflammatory cytokines provides a mechanism as biomarkers for preterm birth and other complications [27]. Given the associations between pregnancy-specific anxiety and poor birth outcomes, it is critical to assess the longitudinal changes in these markers both independently and in relation to psychosocial measures of anxiety as well as association with interventions focused on maternal anxiety and depression. Within the same M-O-M-S™ patient sample, we reported elsewhere that anxiety, associated with *Preparation for Labor*, increased the odds of preterm birth by 60% [16]. In parallel, the associated biomarker data reflected an increased inflammatory response within the control group for anxiety related to *Preparation for Labor*, which aligns with the reported birth outcomes.

## 5. Strengths and Limitations

While the findings are important, they must be considered in the context of the study’s strengths and limitations. The women participating in the biomarker element of the study were a small fraction of the overall sample of women participating in the RCT. Due to the proximity of the clinical and research laboratories for specimen collection and processing, recruitment was limited to women receiving obstetrical care at one of two military treatment locations. Additionally, over the course of the study, participant obstetrical care transitioned almost exclusively to the non-study site location, limiting recruitment significantly. Finally, cytokine biomarkers were selected based on published literature, and the full complement of Th1, Th2, and Th17 cytokines were not evaluated on the available serum. Instead, IL-6/TNF-α and IL-10 were utilized as representative biomarkers for their perspective pro- and anti-inflammatory function, respectively, and ratios of IL-6 and TNF-α to IL-10 were used to describe their antagonistic pro- and anti-inflammatory relationships.

In human studies, capturing and controlling all the factors that may influence cytokine variation is difficult. The sampling design attempted to control for some of the factors known to influence cytokine variation. Accordingly, pregnant women with a history of diabetes mellitus requiring insulin, thyroid disorders, chronic renal or heart disease, and/or a history of asthma were excluded from the study. Furthermore, there was no history of smoking for any of the women in the study, and none of the women in the biomarker study received steroids during their pregnancies.

The effect of BMI on the biomarkers was assessed in two ways. First as a baseline measure (measured upon entry into prenatal care), and second as a mean over the course of pregnancy (a weight taken at each prenatal appointment). When the baseline measure of weight was used for the BMI vs. the mean over pregnancy, there were no significant associations between BMI and any of the longitudinal biomarker values. When the time-varying nature of BMI over the course of pregnancy was considered, there was a significant association with increases in the trend of IL-6 (*p* = 0.01). It does not appear that BMI confounded our key findings.

## 6. Conclusions

Our results suggest that the protective, balancing effect of pro- and anti-inflammatory cytokines in pregnancy is negatively affected by increased maternal pregnancy-specific anxiety. More importantly, it appears that the M-O-M-S™ early, prenatal support intervention decreased pregnancy-specific anxiety and depressive systems and promoted a unique balance of both pro- and anti-inflammatory cytokines in pregnancy. Of particular interest is the slight variation in the psychosocial measures of pregnancy-specific anxiety that were associated with a relatively dramatic physiologic change, which highlights a more nuanced relationship between anxiety and predicted outcomes. Moreover, the theories of chronic stress and maternal immunity are critical in understanding the downstream effects of pregnancy-specific anxiety. Certainly, any of the psychosocial dimensions of anxiety can develop into chronic stress if not addressed; *Preparation for Labor* and *Relationship with Spouse/Partner* were of particular interest due to their association with IL-6/IL-10 and TNF-α/IL-10 ratios. The findings reinforce the need for early, prenatal intervention. While certain prenatal birthing classes may help alleviate some of the fears and anxiety associated with labor, these classes or programs are generally provided late in the third trimester. Additionally, currently there are generally little to no opportunities to obtain support and reflect on one’s relationships relative to pregnancy and parenthood. Even more important, without intervention anxiety associated with maternal identification will not change [18,19]. Clearly, assessment and focused intervention must occur early in pregnancy to promote a balanced/responsive maternal immune system and improve birth and infant outcomes.

## Figures and Tables

**Table 1:** Summary of sample characteristics.

Variable Name	Total Sample (*n* = 57)	Intervention Group (*n* = 28)	Control Group (*n* = 29)
Age (years, mean (SD))	27.81 (4.50)	26.29 (4.29)	29.28 (4.26)
Age (frequency, %)			
≤25	16 (28.07)	12 (42.86)	4 (13.79)
>25	41 (71.93)	16 (57.14)	25 (86.21)
Race/ethnicity (%)			
White, non-Hispanic	32 (57.14)	18 (64.29)	14 (50)
Black, non-Hispanic	8 (14.29)	2 (7.14)	6 (21.43)
Hispanic	10 (17.86)	5 (17.86)	5 (17.86)
Others	6 (10.71)	3 (10.71)	3 (10.71)
Prior deliveries (%)			
0	18 (31.58)	9 (32.14)	9 (31.03)
1 to 2	35 (61.40)	18 (64.29)	17 (58.62)
3 or more	4 (7.02)	1 (3.57)	3 (10.34)
Marital status (%)			
Married	53 (92.98)	25 (89.29)	28 (96.55)
Not married	4 (7.02)	3 (10.71)	1 (3.45)
Military branch ^1^			
Air Force	42 (75)	21 (75)	21 (75)
Army	7 (12.50)	4 (14.29)	3 (10.71)
Other	7 (12.50)	3 (10.71)	4 (14.28)
Active-duty (%)	20 (35.09)	9 (32.14)	11 (37.93)
Active-duty spouse (%)	51 (91.07)	24 (85.71)	27 (96.43)

^1^ Military branch based on participant’s branch of service unless spouse is active-duty member.

**Table 2:** Descriptive statistics for maternal serum biomarkers by trimester.

	Trimester 1			Trimester 2			Trimester 3		
Mean (*SD*)	Total *N* = 57	MOMS™ *n* = 28	Control *n* = 29	Total *N* = 57	MOMS™ *n* = 28	Control *n* = 29	Total *N* = 57	MOMS™ *n* = 28	Control *n* = 29
IL2	3.40	3.40	3.40	3.39	3.36	3.42	3.46	3.47	3.45
	(0.62)	(0.73)	(0.50)	(0.47)	(0.48)	(0.47)	(0.51)	(0.57)	(0.45)
IL6	0.34	0.36	0.33	0.35	0.34	0.35	0.38	0.38	0.39
	(0.07)	(0.08)	(0.07)	(0.09)	(0.09)	(0.09)	(0.10)	(0.11)	(0.08)
IL10	2.68	4.25	1.16	2.74	3.99	1.53	2.36	3.31	1.41
	(5.19)	(7.07)	(0.93)	(4.26)	(5.73)	(1.23)	(3.19)	(4.03)	(1.62)
IL1b	4.09	4.72	3.49	3.35	3.59	3.12	3.71	3.99	3.44
	(4.21)	(5.44)	(2.47)	(1.43)	(1.89)	(0.72)	(2.45)	(2.68)	(2.20)
TNF-α	23.97	25.31	22.68	18.38	17.25	19.46	19.29	17.98	20.60
	(20.76)	(23.71)	(17.77)	(13.27)	(11.91)	(14.59)	(17.44)	(14.52)	(20.13)
TNF-α/IL10	22.00	15.53	28.24	15.65	12.36	18.82	21.52	12.85	30.18
	(28.01)	(19.77)	(33.31)	(15.90)	(13.60)	(17.48)	(37.44)	(13.38)	(50.20)
IL6/IL10	0.32	0.24	0.40	0.30	0.25	0.35	0.42	0.29	0.55
	(0.21)	(0.20)	(0.19)	(0.22)	(0.22)	(0.21)	(0.39)	(0.27)	(0.44)
CRP	2.55	3.05	2.04	5.23	5.93	4.49	5.53	6.15	4.85
	(3.02)	(3.35)	(2.60)	(2.95)	(3.17)	(2.56)	(2.72)	(2.54)	(2.80)
CORT	9.26	9.39	9.13	13.79	13.28	14.28	20.43	20.65	20.22
	(2.92)	(1.90)	(3.67)	(4.80)	(4.40)	(5.18)	(8.04)	(7.08)	(8.99)
CRH	66.65	77.73	55.96	258.60	266.29	251.16	404.16	414.96	393.74
	(53.13)	(60.61)	(43.15)	(110.47)	(104.42)	(117.38)	(115.75)	(101.63)	(128.87)

**Table 3:** Longitudinal relationship of TNF-α/IL-10 and Preparation for Labor and Relationship with Spouse/Partner for the control group.

Model Type	Predictor	Intercept	Coefficient	*p*-Value
Univariate model	Preparation for Labor	52.47 (12.70)	−2.08 (0.94)	0.03
Univariate model	Relationship with Spouse/Partner	57.81 (13.04)	−2.85 (1.10)	0.01
Combined model		73.14 (15.76)		
	Preparation for Labor		−1.61 (0.95)	0.10
	Relationship with Spouse/Partner		−2.42 (1.12)	0.04

## Data Availability

The data is not available for public use but requests for use of de-identified data can be made to the corresponding author.
